# The Acute Effect of Hyperoxia on Onset of Blood Lactate Accumulation (OBLA) and Performance in Female Runners during the Maximal Treadmill Test

**DOI:** 10.3390/ijerph18094546

**Published:** 2021-04-25

**Authors:** Thays C. Silva, Felipe J. Aidar, Aristela de Freitas Zanona, Dihogo Gama Matos, Danielle D. Pereira, Paulo Emmanuel Nunes Rezende, Alexandre Reis Pires Ferreira, Heleno Almeida Junior, Jymmys Lopes dos Santos, Devisson dos Santos Silva, Felipe Douglas Silva Barbosa, Mabliny Thuany, Raphael F. de Souza

**Affiliations:** 1 Department of Physical Education, Federal University of Sergipe (UFS), São Cristóvão, Sergipe 49100-000, Brazil; thaysc.silva@hotmail.com (T.C.S.); fjaidar@gmail.com (F.J.A.); pauloemmanuel92@gmail.com (P.E.N.R.); devissonedfisica@gmail.com (D.d.S.S.); 2 Group of Studies and Research of Performance, Sport, Health and Paralympic Sports (GEPEPS), Federal University of Sergipe (UFS), São Cristóvão, Sergipe 49100-000, Brazil; dihogogmc@hotmail.com (D.G.M.); danielle.dutra@ufpe.com (D.D.P.); 3 Post-Graduation Program of Physical Education, Federal University of Sergipe (UFS), São Cristóvão, Sergipe 49100-000, Brazil; jymmys.lopes@gmail.com (J.L.d.S.); mablinysantos@gmail.com (M.T.); 4 Department of Occupational Therapy, Federal University of Sergipe—UFS, Sergipe 49100-000, Brazil; aristela.zanona@ufs.br; 5 Department of Physiology and Pharmacology, Federal University of Pernambuco, Recife, Pernambuco 50670-901, Brazil; 6 College of Physical Education, University of Brasília–UnB, Brasília 70910-900, Brazil; alexandrerpf92@gmail.com; 7 Post Graduate Program in Physiology Sciences, Department of Physiology, Federal University of Sergi, pe—UFS, São Cristóvão 49100-000, Brazil; heleno.taichi@gmail.com; 8 Post Graduate Program in Health Sciences, Federal University of Sergipe—UFS, Sergipe 49100-000, Brazil; felipedouglas@live.com

**Keywords:** anaerobic, hyperoxia, running

## Abstract

The objective of this study was to analyze the acute effect of hyperoxia during the maximal treadmill test (MTT) of runners. Participants included 10 female street runners who performed the MTT under two different conditions: hyperoxia (HYPX), inhaling oxygen (60% O_2_) every 3 min; and normoxia (NORM), without additional oxygen inhalation. Both groups performed the MTT with increases in the slope of the run every 3 min until voluntary exhaustion. The variables of lactate concentration, the onset of blood lactate accumulation (OBLA), peripheral oxygen saturation (SpO_2_), heart rate (HR), and Borg scale were evaluated. It was verified after the comparison (HYPX vs. NORM) that stage 3 (*p* = 0.012, Cohen’s d = 1.76) and stage 4 (*p* < 0.001; Cohen’s d = 5.69) showed a reduction in lactate under the HYPX condition. OBLA under the HYPX condition was identified at a later stage than NORM. There were no differences in Borg scale, SpO_2_, and HR between the different conditions. It was concluded that the HYPX condition contributed to a reduction in lactate concentration and delayed OBLA in runners.

## 1. Introduction

Aerobic exercise performance has been shown to be greatly mediated through modifications in the arterial content of oxygen. However, the efficiency of the use of oxygen-enriched air (O_2_), improving performance through increasing oxygen delivery, remains controversial [1,2]. On the one hand, the use of hyperoxia has increased in popularity during race training in an attempt to improve performance [1,2] but, contradictorily, hyperoxia may also decrease muscle blood flow during rest from exercise in the forearm [3] and leg [4].

Hyperoxia is defined as the inspiration of oxygen above the partial pressure found in atmospheric air (21% at a barometric pressure of 760 mmHg at sea level) [5]. Favorable indications of hyperoxia after inhalation include respiratory rate reduction, heart rate (HR), muscle glycogenolysis [6], an increase in muscle power [7], presence during exhaustion [8], blood flow distribution [9], and the maintenance of O_2_ saturation [10].

The effect of hyperoxia as an ergogenic resource is a current practice investigated by the World Anti-Doping Agency [6] and is of great interest to athletes seeking to improve their performance. Athletes can weight train with large workloads or prolong the duration of effort when using hyperoxia.

Studies that evaluated intermittent hyperoxia with periods of low O_2_ saturation (values below normobaric conditions of 21% O_2_) have shown an acceleration in muscle recovery, which is considered to be beneficial for plasma membrane resistance, antioxidant capacity [11], increased serum erythropoietin synthesis, and muscle oxygenation [12]. On the other hand, although hyperoxia is proven to be related to metabolic reflexes, insufficient evidence has been presented related to O_2_ administration in physical training [6]. While studies have shown beneficial effects of hyperoxia on athletic performance [13,14], there is still no consensus, especially about using a fraction of inspired oxygen ≥ 0.30 [2] and monitoring lactate concentration [15].

The monitoring of lactate concentration under hyperoxia has been extended to other sports given its applicability by coaches and athletes in cases of continuous exercise prescription in evaluation of the onset of blood lactate accumulation (OBLA). Substantial studies in humans have examined the effects of hyperoxia using blood lactate [4,10,16,17,18], however, there is evidence that during incremental exercise [19] or in knee extensors and exercise [18], there was no effect of hyperoxia on lactate release at either submaximal or peak work rates [20].

However, it has proposed that decreased blood lactate with hyperoxia may occur due to decreased glycogenolysis, glycolysis and ultimately, reduced pyruvate production [21]. Doubt still exists as to what limits the rate of oxidative phosphorylation during aerobic exercise [15]. The common viewpoint proposes that increased lactate formation with increasing exercise intensities is caused by an imbalance between O_2_ supply and demand, culminating in muscle hypoxia.

A recent study reviewed the effect of hyperoxia on performance, training, and recovery, however, overall, hyperoxia had a small effect on decreasing lactate build-up during maximal exercise in time to exhaustion tests, time trials, and graded exercise tests [2]. The literature is inconclusive.

The present study aimed to evaluate the acute effect of the use of hyperoxia (60%) during the maximal treadmill test (MTT), hypothesizing that runners after a hypersaturated inhalation of O_2_ present a lower lactate concentration and a later OBLA during races, submaximally, when compared with the normobaric state.

## 2. Materials and Methods

### 2.1. Design and Sample

Overall, 10 female street runners (age, 28.1 ± 9.2 years; height, 1.61 ± 0.05 m; weight, 58.3 ± 7.2 kg), who were members of the Federal University of Sergipe (UFS) Racing Club, participated in this study. The participants were selected by the criteria mentioned below and signed an informed consent form and received an explanation. This study was submitted for approval by the Research Ethics Committee of the Health Sciences Sector of the UFS (CAAE-60311816.1.0000.5546).

The following inclusion criteria were used: runners with a pace of 6–7 min/km, participants in street races (5 km), who had suffered no lesions in the lower limbs in the last five months. Volunteers who had started another physical activity between the test and the retest period or during the performance, or those who felt any symptom of malaise, nausea, or dizziness, were excluded from the study.

The adopted procedures followed the norms of ethics in research with humans according to Resolution no. 466 of 12 December 2012, of the National Council of Health, conforming to research involving human beings, in agreement with the ethical principles contained in the Declaration of Helsinki (1964, reformulated in 1975, 1983, 1989, 1996, 2000, and 2008) of the World Medical Association.

### 2.2. Protocol

This study was carried out in the Physical Education Laboratory of the UFS (at sea level) in the morning. All volunteers were acclimated and resided in the city of Aracaju, Sergipe, Brazil. Participants were randomly assigned to 2 different regimens for running: under normoxia (normal oxygen pressure) (NORM) or under hyperoxia (HYPX) (60% O_2_) conditions. The tests were carried out on 2 different days with 1-week interval. On the first visit, they were familiarized with the use of the mask. Then, they started the MTT, according to the first regime (HYPX or NORM) which was randomized. On the second visit, the participants returned to the laboratory to perform the MTT under the second condition (HYPX or NORM). All volunteers were not aware of the hypothesis of the investigation and ran under both conditions.

**Figure 1:**
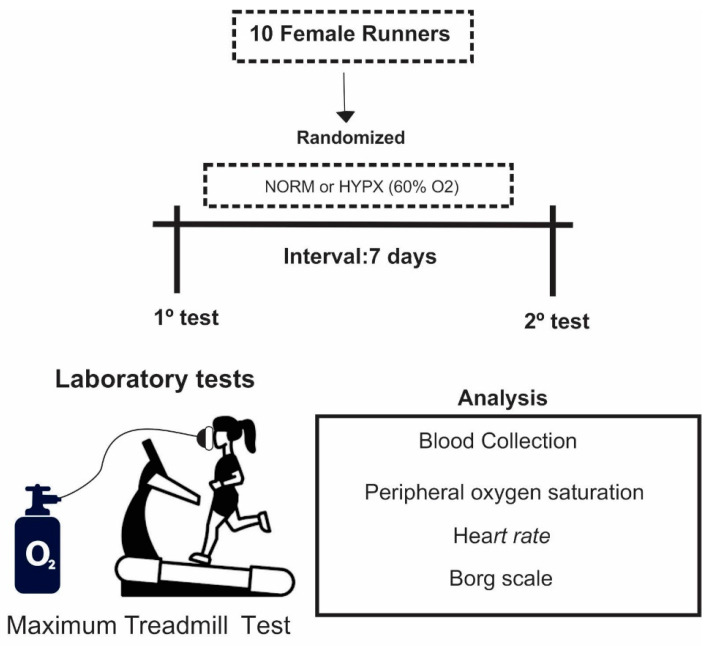
Experimental design. HYPX: hyperoxia; NORM: normoxia.

*Hyperoxia protocol:* During the MTT, a mask system coupled with non-rebreathing oxygen masks (AirLife High Concentration Oxygen Masks, Carefusion 303, Inc., Vernon Hills, IL, USA) connected to an oxygen cylinder was used (99.5% White Martins, Pernambuco). O_2_ was inhaled continuously during each 3 min running stage (Figure 1).

*Research design*: One week prior to the MTT, all participants were present in the laboratory for instructions and anthropometric data collection. To familiarize the participants, a treadmill run of 5 min was performed, and the procedure for using the oxygen mask was explained.

*Maximum treadmill test*: The MTT was performed using the Bruce protocol [10] applied on a treadmill (Kikosks 5403, 110 V, São Paulo, SP, Brazil). The test started at a speed of 2.7 km/h and a slope of 10%, and there were increments of 1.3 km/h and 2% every 3 min until voluntary exhaustion. At each stage, heart rate (HR), peripheral blood saturation (SpO_2_), lactate (Lac), and Borg scale were monitored. OBLA is the workload or metabolic rate at which blood lactate concentration begins to increase exponentially. It has been shown to correspond to approximate blood lactate of 4 mmol/L [22,23]. Before the test, all the volunteers underwent a warm-up of 15 min of running at low intensity (70% HR), followed by general stretching exercises. The test was completed when the participant expressed verbally the impossibility of continuing to run due to muscular fatigue.

*Blood collection*: For the collection of blood, an Accuchek lancet was used with disposable lancets and the distal phalanx of the index finger was perforated. The device used was an Accutrend Lactate Accu-Check lactometer (Roche, Rotkreuz, Switzerland) with BM-Lactate reagent strips. Blood collection was performed during the run every 3 min. Before perforation, the fingers of the athletes were cleaned with cotton soaked in alcohol 90%. Approximately 25μL blood were collected. The blood sample was placed directly on the test strips. The establishment unit for analysis was millimoles per liter (mmol/L).

*Peripheral oxygen saturation and heart rate*: SpO_2_ measurement was performed using the Dixtal model Superbright-DX 2455 (Philips, Amsterdam, the Netherlands) with a sensor positioned on the third finger of the right hand, with the reading being determined after signal stabilization. At the same time, HR was determined. The devices have a receptacle to accommodate the distal portion of the finger, with one side containing a light source, composed of two light-emitting diodes (LEDs), and on the other side, a photodetector. One LED emits red light (≅660 nm) and another emits infrared light (≅940 nm). The measured values of maximum heart rate (HRmax) were estimated by the equation “207 − 0.7 × age” [24].

*Borg scale*: The effort perception scale was recorded every 3 min according to the Borg category rating scale [25]. This scale consists of an enumeration of 0–10, in which the value 0 indicates an absence of perception of physical fatigue and 10, extreme physical fatigue [7]. The Borg scale was explained to the participants before performing the requested test and applied every minute after the start for the runner to indicate the level of perceived effort.

### 2.3. Statistical Analysis

To verify the distribution of the sample, a normality and homogeneity test was carried out using the Shapiro–Wilk and Levene tests. The analysis of the possible differences between the conditions was performed by an ANCOVA test using dependent variables (lactate, heart rate, Borg scale, and SpO_2_) × fixed factor (experimental condition) × covariate (Bruce test stages) followed by a Bonferroni post hoc test. To assess the effect size, the Cohen’s d test was used, adopting the cut-off points of 0.02–0.15 as small effect, 0.16 to 0.35 as medium effect, and greater than 0.35 as large effect [26]. The relationship between lactate concentration and heart rate was verified by Pearson’s correlation. The magnitude of the correlation was determined by the scale proposed by Batterham and Hopkins [27], as follows: r < 0.1, trivial; r = 0.1–0.3, small; r = 0.3–0.5, moderate; r = 0.5–0.7, strong; r = 0.7–0.9, very strong; r = 0.9–0.99, almost perfect; and r = 1.0, perfect. The 95% confidence intervals (95% CI) were computed. We performed a statistical power analysis to estimate the appropriate number of participants required to generate these results. Using G Power program (3.1), we calculated an effect size (f = 0.9) with 60% confidence (power = 0.6) and α err prob (0.05) in ANOVA with blocking and replication. The differences between the means with a *p*-value < 0.05 were considered statistically significant. The data were analyzed using SPSS software 20.0 (IBM, North Castle, New York, NY, USA).

## 3. Results

**Figure 2:**
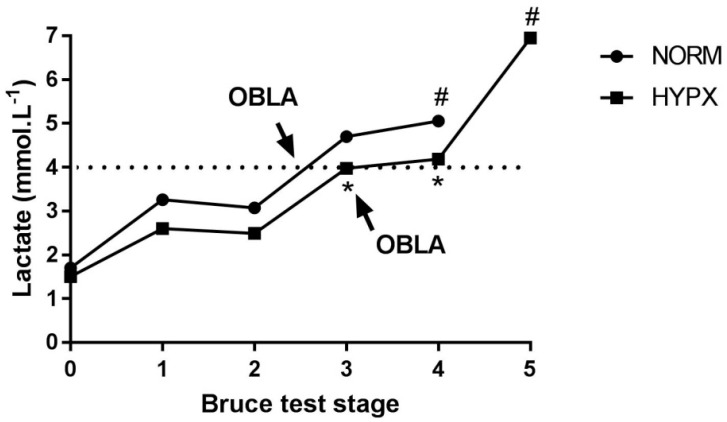
Stage of lactate. HYPX: hyperoxia; NORM: normoxia; OBLA = onset of blood lactate acumulation; * HYPX vs. NORX; ^#^ stage 1 vs. stage 4 and 5 MTT; *p* < 0.05.

At the end of the MTT, it was possible to observe that the HYPX condition presented positive effects for performance, reaching a running stage superior to NORM. HR did not show any significant difference between NORM vs. HYPX under any of the conditions (*p* > 0.05) (Table 1). The lactate (Figure 2) concentration during the MTT under HYPX condition was lower in stage 3 (3.9 ± 0.4 vs. 4.7 ± 0.5; *p* = 0.012 Cohen’s d = 1.76) and in stage 4 (4.1 ± 0.2 vs. 5.0 ± 0.1; *p* < 0.001 Cohen’s d = 5.69).

**Figure 3:**
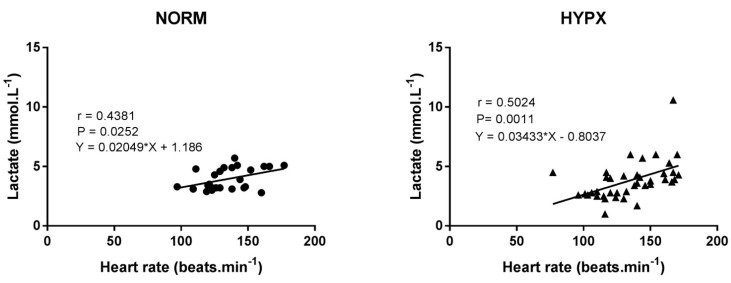
Correlation coefficients between lactate concentration and heart rate. HYPX: hyperoxia; NORM: normoxia.

Correlation coefficients between lactate concentration and heart rate under HYPX show a moderate effect (r = 0.5024; 95% CI = 0.2221 to 0.706; *p* = 0.0011), and under NORM the effect was small (r = 0.4381; 95% CI = 0.06111 to 0.7057; *p* = 0.0252) (Figure 3).

**Figure 4:**
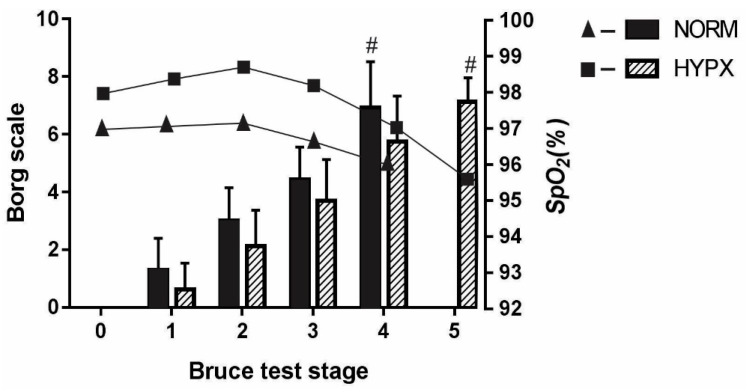
Stage of Borg scale and stage of peripheral oxygen saturation. HYPX: hyperoxia; NORM: normoxia; SpO_2_ = peripheral oxygen saturation ^#^ stage 1 vs. stage 4 and 5 MTT; *p* < 0.05.

The Borg scale did not indicate any significant difference between NORM vs. HYPX in the pre-test conditions (*p* = 0.13; Cohen’s d = 0.81), or in the post-test (*p* = 0.79; Cohen’s d = 0.15). Similarly, peripheral blood oxygen saturation also did not show differences (*p* = 0.59; Cohen’s d = 0.01 and *p* = 0.56; Cohen’s d = 0.01, respectively) (Figure 4).

## 4. Discussion

This study evaluated the acute effect of hyperoxia on the performance of 5 km runners, hypothesizing that the hyperoxic state could be beneficial for physical performance, which was identified as favorable after the analysis of the results of lactate concentration.

At the end of the MTT, the concentrations of lactate were found to be greater than 4 mmol.L^−1^ under both NORM and HYPX conditions. This result can be considered physiologically normal due to the gradual difficulty imposed by the test. The increase in blood and muscle lactate resulted from the acid–base imbalance, decreasing the pH due to the production of lactic acid and to metabolic stress [28].

On the other hand, the low concentration of lactate found under the HYPX condition in stages 3 and 4 reinforces the fact that the production of lactate during exercise is dependent on O_2_ [21]. Thus, the OBLA is influenced, as identified more strongly during the MTT with maximal exercise, where the athlete achieves a stage above the NORM condition.

Thus, the higher metabolic stress observed in the NORM condition was also related to marked muscle fatigue after reaching OBLA, as observed in Figure 2, when the blood lactate concentration increased exponentially, accelerating the withdrawal from the evaluation process [6]. These findings corroborate other evidence proposing that the acute effect of HYPX may favor sport performance. Spriet et al. [29] and Sperlich et al. [6] discussed the economics of adenosine triphosphate (ATP) in the glycolytic flow [29] and a decreased production of pyruvate [6]. Furthermore, Hogan et al. [21], when evaluating the effect of up to 60% of O_2_ inhalation during physical activity in an ergometer cycle, found that lactate concentrations were significantly lower during the state of hyperoxia and higher during hypoxia compared to normoxia. However, there was no change in the lactate values regarding exhaustion between the three treatments.

Moreover, some records suggest that leg blood flow is reduced during hyperoxic exercise [30]. Evidence indicates that erythrocytes have the ability to sense changes in O_2_ and to modulate vascular tone (via release of ATP and/or nitric oxide), providing appropriate changes in blood flow and O_2_ delivery with metabolic need [31]. Under normoxic conditions and hemoglobin desaturation, the responsiveness of vasodilators increases, so ATP is released from erythrocytes and is thought to contribute to the augmented blood flow [32]. Still, in response to large increases in O_2_, an attenuated release of ATP may have blunted the blood flow response to exercise [3].

The analysis of HYPX regarding the performance during the MTT also was verified through the correlation of lactate concentration and heart rate. Under the HYPX condition, there was a moderate relationship, however, it was substantially greater than in the NORM condition. In fact, while the blood lactate concentration tends to increase when the equilibrium between production–removal is gradually reduced, the cardiovascular system tends to readjust, apparently with a higher ratio under the HYPX condition, after the stressful stage.

In our study, we did not perform specific assessments on effects related to blood flow, given that there was no reduction in HR under HYPX reported in previous studies [33,34]. However, the fact that we used substantially less exposure to the gas was observed with the result that hyperoxia induced alterations in cardiovascular function and autonomic control between 45 min [34] and 1 h [33]. Furthermore, it has been mentioned that ATP was responsible for a part of the muscle vasodilation that followed muscle hyperemia during exercise [35]. On the other hand, we can relate our results to other reasons, probably attributable to the increase in microvascular and intracellular oxygenation during exercise when compared to normoxia [36,37]. In addition, Goulding et al. [36] demonstrated an increase in the concentration of muscle oxyhemoglobin under the condition of hyperoxia, which is an important physiological determinant for increasing exercise extension and tolerance.

Even though acute exposure to HYPX may have performance benefits, chronic exposure has been reported as potentially harmful to health, depending on the duration of application [6]. This is related to oxidative stress and consequent cellular damage or dysfunction, due to the formation of reactive oxygen species, increasing the probability of myocardial infarction [15,28]. Prompt monitoring of blood markers during exercise and athlete recovery has been suggested [38]. Similarly, continuous exposure of rodents to hyperoxia (72 h) resulted in cardiotoxicity and cardiac arrhythmias [39].

Stability of the SpO_2_ patterns was maintained under the HYPX condition, interpreted as resulting from the increase in intracellular vascular gradients, suggesting the occurrence of an increase in the oxygen supply to the capillaries of the muscle cells, and, consequently, O_2_ diffusion to the mitochondria [1]. According to Nummela et al. [40], this analysis may have different results, according to the physical fitness level of the runner; they reported that the process of desaturation is proportional to the intensity of the exercise verified in athletes with better aerobic conditions and greater oxidative capacity when compared to untrained individuals [1].

An average low in SpO_2_ of 91% in NORM is not usual in healthy subjects under the Bruce protocol [41]. This desaturation, however, is compatible with trained endurance athletes, and women seem more prone to this, provided that they have an average VO2max of at least 56–57 mL/kg/min [42,43].

When the Borg scale and HR were analyzed during the MTT, no changes were identified between O_2_ saturation levels in the two conditions, corroborating previous studies [1]. On the other hand, evidence was found indicating that HYPX favors HR recovery after interval training, including reducing blood pH [8]. Thus, we suggest the need for carrying out specific studies in order to better understand the behavior of HR under the HYPX condition, not only during the test but also in the preceding periods.

The fact that evidence has begun to reinforce the positive effect of acute O_2_ supplementation, immediately enhancing performance, increases the questioning of its choice as an ethical strategy or not at sporting events [6]. There remain concerns whether hyperoxic training definitely improves performance and should be banned by the World Anti-Doping Agency. Furthermore, although breathing O_2_-enriched air is commonly used in sport activity at high altitude sites, this practice requires special accessories (facial masks, bottles, or even bags), that cannot normally be carried or used during a sporting event.

Moreover, this practice has also been used as a long-term training stimulus or recovery intervention between exercise sessions. This is despite the fact that there is still a lack of definition in the literature regarding the complexity of the biological responses to hyperoxia: the different methodologies (e.g., exercise intensity and modality, level of oxygen, number of participants), muscles involved (arms and legs), training status of the participants [13], training regimen, type of exercise, and recuperative protocols [2].

Despite the relevance of the findings, the present research has the following limitations, especially considering the small size of our sample. We believe that effect size estimates can be designed specifically to contribute to and characterize results by discussing the magnitude of an effect, in addition to estimates of probability (5%). Therefore, Cohen’s d (1.76 and 5.69 for lactate concentration at stages 3 and 4, respectively), strengthens the evidence of the reduction of the lactate concentration under HYPX. In addition to this, all participants could have been blinded to the supply of O_2_ and the mask system could have also been used for the NORM testing. The evaluation of the VO2 max test could have broadened our analysis, mainly on performance. We noted that the volunteers who underwent the stage 5 Bruce test requested to leave this stage quickly.

Although graded exercise tests are the most commonly used exercise protocols in laboratories, the Bruce protocol may have limitations. On the other hand, this test aims to recreate circumstances during which physiological signs arise. That way, lactate assessment during exercise can provide valuable considerations regarding the athlete’s training level [44]. All of this suggests that new studies are needed with a greater sampling power and stricter parameters.

### Future Applications

Athletic success depends on cardiovascular changes and on oxygen extraction. The performance of aerobic exercise appears to be largely mediated by changes in arterial oxygen content and subsequent oxygen supply. Although we have shown that running under conditions of hyperoxia can improve OBLA, reflecting benefits for performance with greater tolerance to workload, we suggest new studies in order to deepen the diversity of training methodologies (steady state, interval, mixture of hyperoxy and normoxic training), together with differences in fractions of inspired oxygen and duration of exposure to training. In addition, it is difficult to formulate a consensus on the effectiveness of training under hyperoxia until a better understanding of its negative effects (e.g., increased oxidative stress, cell damage or dysfunction).

## 5. Conclusions

We evidenced a substantial improvement under a hyperoxia (60% O_2_) protocol performed by female runners during the maximal exertion test, with OBLA being improved when compared to the state of NORM. We suggest that the strategies of hyperoxia as ergogenic resources should be further investigated under new conditions during the period of race training, interval protocols, and recovery.

## Figures and Tables

**Table 1:** Stage of heart rate.

Condition	Stage 1 (Beats.min^−1^)	Stage 2 (Beats.min^−1^)	Stage 3 (Beats.min^−1^)	Stage 4 (Beats.min^−1^)	Stage 5 (Beats.min^−1^)
HYPX	108.2 ± 8.8	128.7 ± 11.6	144.7 ± 29.7 *	139.7 ± 21.9 *	152.2 ± 15.5 *
NORM	118.8 ± 16.1	133.8 ± 15.3	134.1 ± 13.6	155.8 ± 18.3 *	-

HYPX: hyperoxia; NORM: normoxia; * *p* < 0.05 vs. stage 1.

## Data Availability

The data are not publicly available due to ethical concerns.
